# Anti-inflammatory loaded poly-lactic glycolic acid nanoparticle formulations to enhance myocardial gene transfer: an in-vitro assessment of a drug/gene combination therapeutic approach for direct injection

**DOI:** 10.1186/1479-5876-12-171

**Published:** 2014-06-16

**Authors:** Anthony S Fargnoli, Anbin Mu, Michael G Katz, Richard D Williams, Kenneth B Margulies, David B Weiner, Shu Yang, Charles R Bridges

**Affiliations:** 1 Thoracic and Cardiovascular Surgery, Sanger Heart & Vascular Institute, Carolinas Healthcare System, Charlotte, NC, USA; 2 Internal Medicine, Perlman School of Medicine, University of Pennsylvania Medical Center, Philadelphia, PA, USA; 3 Pathology and Laboratory Medicine, Perlman School of Medicine, University of Pennsylvania Medical Center, Philadelphia, PA, USA; 4 Materials Science Engineering, School of Engineering & Applied Science, University of Pennsylvania, Philadelphia, PA, USA

**Keywords:** Combination therapy, Cardiac gene delivery, Nanotechnology, Myocyte expression

## Abstract

**Background:**

Cardiac gene therapy for heart disease is a major translational research area with potential, yet problems with safe and efficient gene transfer into cardiac muscle remain unresolved. Existing methodology to increase vector uptake include modifying the viral vector, non-viral particle encapsulation and or delivery with device systems. These advanced methods have made improvements, however fail to address the key problem of inflammation in the myocardium, which is known to reduce vector uptake and contribute to immunogenic adverse events. Here we propose an alternative method to co-deliver anti-inflammatory drugs in a controlled release polymer with gene product to improve therapeutic effects.

**Methods:**

A robust, double emulsion production process was developed to encapsulate drugs into nanoparticles. Briefly in this proof of concept study, aspirin and prednisolone anti-inflammatory drugs were encapsulated in various poly-lactic glycolic acid polymer (PLGA) formulations. The resultant particle systems were characterized, co-delivered with GFP plasmid, and evaluated in harvested myocytes in culture for uptake.

**Results:**

High quality nanoparticles were harvested from multiple production runs, with an average 64 ± 10 mg yield. Four distinct particle drug system combinations were characterized and evaluated in vitro: PLGA(50:50) Aspirin, PLGA(65:35) Prednisolone, PLGA(65:35) Aspirin and PLGA(50:50) Prednisolone Particles consisted of spherical shape with a narrow size distribution 265 ± 104 nm as found in scanning electron microscopy imaging. Prednisolone particles regardless of PLGA type were found on average ≈ 100 nm smaller than the aspirin types. All four groups demonstrated high zeta potential stability and re-constitution testing prior to in vitro. In vitro results demonstrated co uptake of GFP plasmid (green) and drug loaded particles (red) in culture with no incidence of toxicity.

**Conclusions:**

Nano formulated anti-inflammatories in combination with standalone gene product therapy may offer a clinical solution to maximize cardiac gene therapy product effects while minimizing the risk of the host response in the inflammatory myocardial environment.

## Background

Acquired heart disease from myocardial infarction (i.e. heart attack) remains the leading cause of mortality and morbidity worldwide, with 22 million new patients diagnosed annually. Essentially, all approved pharmacologic and device systems impose significant cost burden to the health system, yet fail to increase survival rates [1-3]. Heart transplant, which is the gold standard for patients, will never meet clinical demand due to the chronic shortage of viable donors [4,5]. Therefore, new therapeutic approaches to manage the disease burden represent a significant unmet need. Recently, sophisticated molecular profiling tools combined with a deeper knowledge base derived from disease models have ushered in a new era of biopharmaceutical development for heart disease. This has resulted in the development of a more potent class of therapies designed to act at the myocyte level, whereby therapeutic action is achieved primarily through DNA, RNA and or microRNA genetic reprogramming [6]. Various gene therapy concepts have been applied successfully in animal models demonstrating increased contractility, repaired myocardium, and or regenerated new vessels to reduce myocardial infarction reoccurrence [7-10]. Independent of the targeted gene mechanism, the most common means to achieve these aims are with either bioengineered viral or non-viral vector biologics, since the uptake and success rates of naked molecular therapies is very poor in vivo [11,12]. The most effective gene products today have shown remarkable promise, but at the same time have also presented more risks and complicated translational issues, especially when compared with traditional pharmaceutical compounds.

Despite the availability of effective transgene-vector systems, one major rate limiting problem is with achieving safe and efficient myocardial gene transfer in the clinic [13,14]. Due to size scale and more complex membrane barriers, these issues do not emerge in smaller animal studies yet are a major challenge in larger organisms [15,16]. Although the preferred route of administration in clinical trials, it remains controversial whether or not minimally invasive catheter infusion approaches can yield sufficient therapeutic expression levels that significantly improve outcomes in the clinic [17]. Another major problem with these systems is restricting therapeutic expression to the heart and minimizing off target effects. In fact, published large animal data has demonstrated a greater than 2000 fold higher presence in collateral organs versus the heart [18-20]. Alternatively, direct myocardial delivery methods can restrict therapeutics to the heart if safely administered.

Direct myocardial delivery methods (e.g. needle injection, sonoporation) can offer greater cardiac specificity of gene therapeutics compared to percutaneous infusion approaches. The key unresolved problem is with the limited distribution of gene therapeutic per delivery site requiring many injections [21]. Increasing the number of injections has the adverse effect of triggering inflammation in the myocardium, thus limiting the availability of additional injection sites and jeopardizing the retained therapy. The immune response to gene therapy products, especially notorious with the viral mediated products, is complex but several key studies have demonstrated a clearer relationship between inflammation and the increased risk of an adaptive immune response [22,23]. Therefore it is postulated the use of an anti-inflammatory drug co-delivered with the gene therapy product could: (1) Address the inflammation to minimize the adaptive immune response and promote therapeutic tolerance (2) increase trafficking and uptake in a more favorable microenvironment and (3) potentially permit more injection sites.

This concept of a direct injection drug/gene approach has yet to be translated into the heart, whereby problems exist with increasing uptake and extending the half-life of anti-inflammatory drugs at the site of injection beyond the peak acute inflammatory window of 48 hours. In addition to the timing issue, the anti-inflammatory load must not interfere with vector trafficking or the subsequent gene expression efficiency. Numerous studies have explored of advanced non-viral vectors to increase in vivo performance by means of transfection alone [24,25]. However, none have attempted to use anti-inflammatories at the injection site co-delivered with a higher risk, but optimal gene transfer vector to provide a more promising clinical strategy. This study summarizes the development and parameter testing of a reliable nanoscale anti-inflammatory formulation production process for co-delivery with gene products. The development phase features aspirin and prednisolone, two widely utilized anti-inflammatories and incorporates them into two common FDA approved poly lactic glycolic acid (PLGA) polymers [26]. Complete nanoparticle characterization, process tolerance limits and an in vitro feasibility assessment in harvested myocytes are offered to evaluate the concept of a drug/gene combination therapy.

## Methods

### Poly-lactic glycolic acid nanoparticle production process

**Figure 1:**
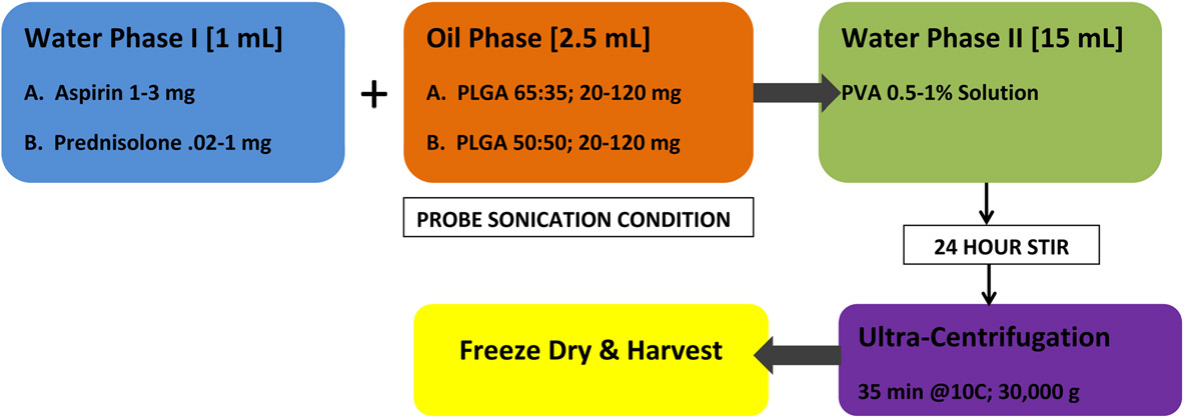
The water oil water double emulsion nanoparticle production process work flow sequence to generate high quality anti-inflammatory formulations.

Pre-Processing Steps:A water oil water (w/o/w) double emulsion process outlined in (Figure 1) was executed to generate aspirin (99% pure, Sigma Aldrich USA) and prednisolone (99% pure, Sigma Aldrich USA) loaded poly- lactic glycolic acid (PLGA) nanoparticles (NPs). First, initial drug load water phase stocks of 1–3 mg/mL aspirin and 0.1-0.4 mg/mL prednisolone were created by dissolving in 1% poly vinyl alcohol (PVA) solution. In the case of prednisolone due to its poor water solubility, a 10% Ethanol (w/w%) was added. These doses were selected based on body weight and pharmacokinetic data for the rodent species. The second step or oil phase was generated in a separate vial, with PLGA, input mass range (20–120 mg) of one of either types (50:50, 65:35 i.e. % of lactic: glycolic acid chains) dissolved in 2.5 mL of Dichloromethane. For the in vitro study only, production runs were carried out as described in the methods above except 100 μg of Rhodamin B dye powder was added to the first drug water phase.

Process Steps:

The first emulsion was created by adding 1 mL of aspirin or prednisolone drug PVA 1% solution dropwise to the oil phase polymer in a 5 mL glass vial under probe sonication. After 3 minutes, this resultant emulsion was then added dropwise to a larger outer water phase containing 15 mL of PVA 1% to create the double emulsion. The double emulsion was then placed in a fume hood and stirred gently for at least 24 hours to facilitate solvent evaporation and particle formation. Separation was achieved with via ultra-centrifugation at 30,000 g for 35 min at 10C. The resultant particle pellets were washed to remove residual drug/polymer, then freeze dried overnight. Four nanoparticle compositions were generated with the reaction: PLGA (50:50 Aspirin), PLGA (65:35 Aspirin), PLGA (50:50 Prednisolone) and PLGA (65:35 Prednisolone).

Post-Processing:

All yields were weighed then stored in sterile cryovial containers at -20C.

### Scanning electron microscopy (SEM) analysis & characterization

Approximately 5 mg of each freeze dried NP sample was prepared for SEM with gold sputter coating, and then imaged on a JEOL SEM unit at 1.00 kV between 10–20,000 x. Multiple images were taken from separate locations on the field with focus in the range of 1 um to 500 nm.

### Stability testing

#### 24 hour formulation stability test

Saline stability re-constituted particles tests were also conducted. Ten mg of freeze dried nanoparticles were dissolved and probe sonicated in sterile 0.9% saline water and allowed to settle over a 24 hour period. Repeat droplets were first dried and sputter coated for loading into the SEM.

#### Zeta potential colloidal stability measurements

A sample of each particle composition was prepared in water and added to testing cuvettes per manufacturer instructions of the Zetasizer Nano ZS instrument (Malvern Instruments, UK). Triplicate runs were averaged to represent a single data point for multiple samples from the same production lot.

### Controlled release & loading efficiency analysis

A UV–vis spectrophotometer (NanoDrop, National Instruments) was used to generate two separate standard curves for serial dilutions of known drug concentrations. The wavelength consistent with aspirin detection was 275 nm and 235 nm for prednisolone.

#### Drug loading efficiency calculations

To compute loading efficiency, the amount of either aspirin or prednisolone encapsulated in the nanoparticle formulations was determined by measuring the residual amount in the supernatant following centrifugation relative to the initial load in the water phase. Percentage was derived as the mass amount of drug remaining in the supernatant following separation.

#### Controlled release analysis

High quality yields were selected based on the most potent drug formulation of 5 wt. % PLGA aspirin and 1.5% PLGA prednisolone were manufactured per process specifications. Each particle formulation was prepared for controlled release studies as follows: (1) 20 mg of particle was dissolved into 10 mL of 42C (2) Samples for spectrophotometry analysis were removed with a syringe 450 nm filter at 12 hours, 1, 2, 3, 4, 5 days (3) The sample volume was replaced and the process repeated for each interval up until the final point (4) Triplicate UV–vis spectrophotometry measurements against the standard curve for each drug were performed on each sample to determine the percentage released for each run.

### In vitro testing protocol

#### Neo-natal rat cardiac-myocyte harvesting

Day 0 to 3 neonatal pups are used and the pups were euthanatized by decapitation and the heart was immediately removed with forceps. The atria and great vessels were removed and the left ventricular tissue was minced and subjected to a trypsin-based disaggregation procedure in a 6 well plate with ethanol cleaned scissor, rinsed with HBSS with 1% P/S/G, and place in a 50 ml conical tube containing 10 ml of Trypsin solution for shaking (200 rpm) at 37C for 15 min. Cells were then centrifuged at 660 rpm at 4c for 5 minutes. The supernatant was discarded and the cells were re-suspended in 20 ml of media and pre-plate for 1–3 hours in the incubator. Harvested cells were collected with centrifuge spin at 660 rpm for 5 min at room temperature. Cell pellets for experiments were then placed in the culture media and counted using 0.4% Trypsin blue.

#### Plasmid GFP DNA & tagged nanoparticle preparation

A 10 mg master aliquot of eGFP plasmid DNA was obtained from Invitrogen and handled according to manufacturer’s instructions. Under sterile conditions working yields for each well were created with, 6 μg of DNA was diluted into 100 μL of RNAase free water. Separately 10 mg of each of the process output 4 resultant particle systems [PLGA50:50-Aspirin, PLGA50:50-Prednisolone, PLGA65:35-Aspirin, PLGA65:35 Prednisolone] tagged with Rhodamin B was finely crushed and mixed into 20 mL of phosphate buffer saline. To remove residual dye not bound within the particle structure, the particle solution was placed into a dialysis membrane submerged in an outer bath of PBS at 37. Prior to well transfection, 10 μg of each nanoparticle solution in 500 μL was placed in individual aliquots.

#### DNA and nanoparticle well transfection

On the day before transfection, cells were placed in 12 well plates with each well seeded at a density of 500,000 myocytes in 1 mL of DMEM (GIBCO) media without antibiotics. The transfection complexes were then prepared:

Complex 1 – One 6 μg of DNA aliquot was diluted into 100 μL of media in an individual eppendorf vial.

Complex 2- 8 μL of Lipofectamine (Invitrogen) was diluted into 100 μL of media.

Then, complexes 1 and 2 were mixed together and permitted to incubate at room temperature for at least 20 minutes. After 20 minutes the contents of the individual eppendorf yields were then transferred into each well. The plate was gently rocked then placed back in the incubator until the first 24 hour imaging time point.

For the nanoparticle treatment wells as designated, the 500 μL filtered sterile solution was added via syringe to each well with a 450 nm to prevent aggregates from transferring.

#### Follow up fluorescent microscopy

The first set of images was taken at 24 hours post transfection. Media was removed from each well prior to imaging and replaced prior to returning to the incubator. The remaining set of images was taken at the 48 hour time point.

Fluorescence images were acquired using a Nikon Eclipse TE2000-U fluorescence microscope equipped with a Plan Fluor × 20/0.50 objective (Nikon, Tokyo, Japan. Microscope controlling and image processing were carried out using Image-Pro Plus 4.5.1.27 (Media Cybernetics, Bethesda, MD, USA).

#### Statistical analysis

All SEM and nanoparticle characterization data was loaded into GraphPrism software suite for statistical testing. Single way ANOVA was utilized to determine differences in nanoparticle subtypes. Individual paired t-tests were used to compare across individual groups. Bonferroni corrections were applied for significance testing.

## Results

### Process capability

Over 45 nanoparticle production yields were obtained over the development course with the optimal ranges. The process volumes were held in a fixed ratio, featuring water phase #1 at 1 mL, the oil phase at 3 mL, and the outer water phase #2 at 15 mLs. Pilot runs in greater amount adhering to this proportion scale yielded the same quality particles. Briefly, basic guidelines for each process phase.

Water Phase: Aspirin 1–3 mg dissolved in PVA 1% or Prednisolone 0.02-1 mg in 10% ethanol PVA1%. It was noted that adding additional solvents to increase drug load in this phase resulted in failure to maintain particle integrity and stability.

Oil Phase: The process was very flexible in terms of changing the amount of polymer added to the system and was stable in the range of 20 – 120 mg of either PLGA type.

Outer Water Phase: The PVA in the system acts as a vital stabilizer that can be readily increased. Increases beyond 2% tended to inhibit the amount but not the quality of generated nanoparticles. Thus, a working range of 0.5-2% of PVA stabilizer in the outer water phase is suitable for accommodating various drug/polymer complexes with good stability.

### Particle characterization

**Figure 2:**
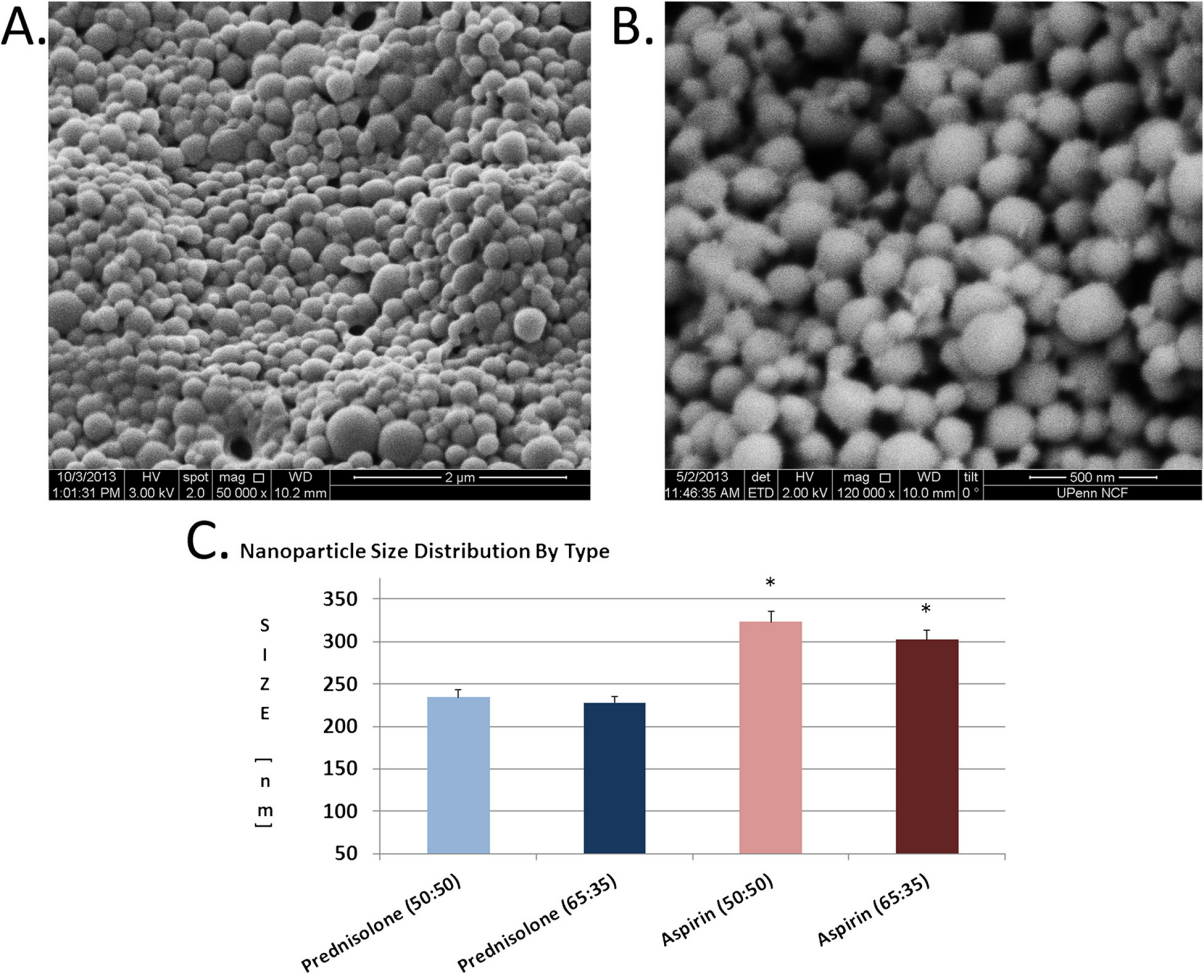
**SEM Characterization Sizing Results. A**. Narrow size distribution and high quality spherical shaped yield example in A at 2μm scaling. **B**. Close up 500 nm scaling image indicates narrow size distribution in the 200-350 nm range consistently for all manufactured yields. **C**. Nanoparticle average size by drug and polymer combination. Aspirin nanoparticles of either 50:50 or 65:35 type had greater size (p<0.05) versus prednisolone.

The results presented here summarize the characterization for each of the 4 resultant nanoparticle types acquired from 5 consecutive runs. Polymer load was fixed at 60 mg, aspirin 3 mg and prednisolone at 1 mg respectively.SEM images from the various runs for each nanoparticle type were loaded into ImageJ software for analysis. The process consistently yields uniform, spherically shaped formulations (Figure 2A,B). The size distribution was very narrow, of high quality and was as follows: PLGA50:50 Prednisolone [234 ± 9 nm], PLGA65:35 Prednisolone [228 ± 7 nm], PLGA50:50 Aspirin [323 ± 13 nm] and PLGA65:35 Aspirin [302 ± 7 nm]. ANOVA indicated significance between the groups, specifically it was determined that aspirin contributed to larger particles as both PLGA50:50 and PLGA 65:35 types were significantly larger than their matched prednisolone counterparts. (Figure 2C) This difference in size was most likely attributable to both higher aspirin mass content and charge of the first water phase in the reaction since size was unaffected by the addition of more polymer (data not shown).

Yields were very consistent and proportional to polymer mass input in the range of 75-80% recovery upon final harvest. The average yields per polymer/drug type based on 60 mg input were: PLGA50:50 Prednisolone [46 ± 1 mg], PLGA65:35 Prednisolone [45 ± 2 mg], PLGA50:50 Aspirin [48 ± 1 mg] and PLGA65:35 Aspirin [47 ± 2 mg]. Production yields with increased or decreased polymer loading revealed the same results (data not shown).

Loading Efficiency results were uniform for all 4 nanoparticle types, independent of drug or polymer and were: PLGA50:50 Prednisolone [88.9 ± 0.01%], PLGA65:35 Prednisolone [88.2 ± 0.01%], PLGA50:50 Aspirin [89.0 ± 0.01%] and PLGA65:35 Aspirin [88.8 ± 0.01%].

### Stability analysis

**Figure 3:**
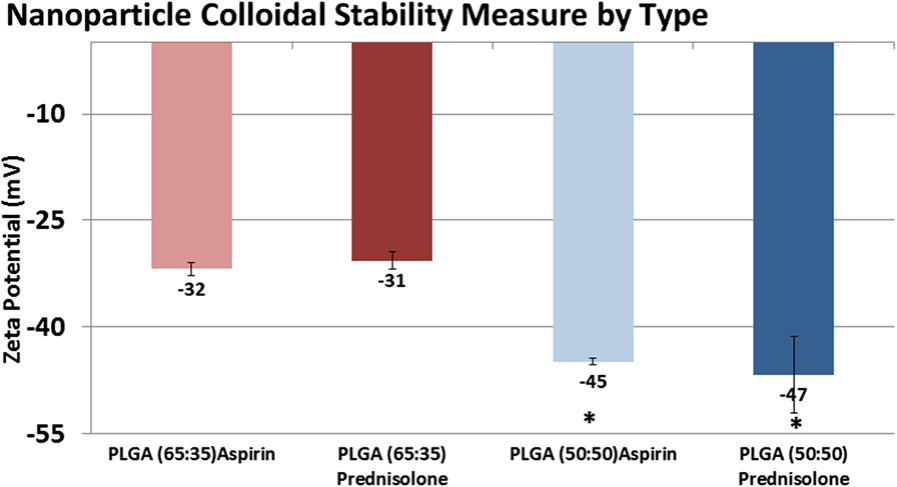
Nanoparticle zeta potential colloidal stability testing results indicate that the PLGA50:50 nanoparticles are more stable in solution versus the PLGA65:35 types.

Positive nanoparticle visualization was realized on the SEM 24 hours after re-constituting freeze dried product in saline for all 4 polymer configurations. The particle shape and size was retained.The stability of the nanoparticles in suspension was moderate to good in the range of -30 to -53 mV. A score much less than -30 indicates a stability issue with a pharmaceutical dispersion, while any score higher than -60 indicates maximum. The potential scores by nanoparticle type shown in Figure 3 were as follows: PLGA50:50 Prednisolone [-47 ± 5 mV], PLGA65:35 Prednisolone [-31 ± 1 mV], PLGA50:50 Aspirin [-45 ± 0.5 mV] and PLGA65:35 Aspirin [-32 ± 0.9 mV]. Statistical tests revealed that the PLGA50:50, independent of drug load was superior compared with the 65:35 type.

### Controlled release of the nanoparticle formulations

**Figure 4:**
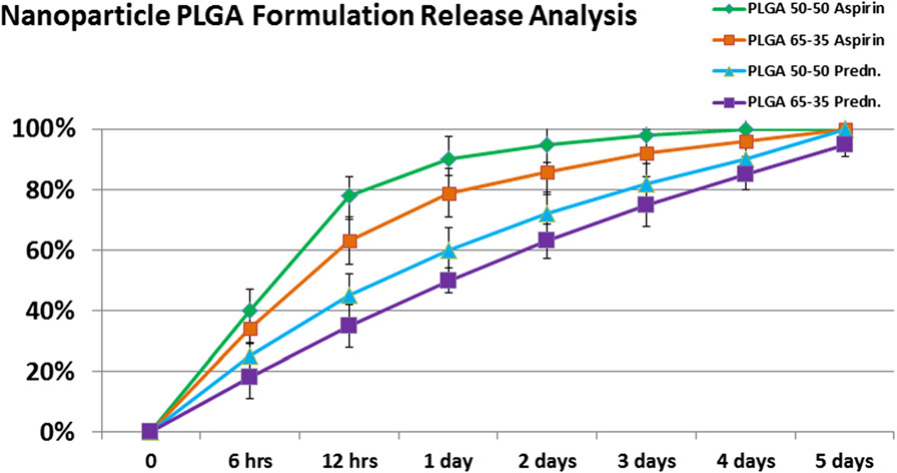
Controlled release study results demonstrate that aspirin particles overall release faster than prednisolone types.

Figure 4 shows a graphical depiction of the release over the span of 5 days. It was evident that the aspirin released faster overall as compared with prednisolone. This is most likely due to a combination of factors including size, stability and charge. The PLGA50:50 Aspirin type had the fastest release profile.

### Process limitations

The high quality in terms of particle shape uniformity, yield, surface charge and release properties were critically limited by a number of key variables. Therefore, production with major deviations with the water phase I input (data not shown) resulted in lower quality profiles featuring aggregation and wider size ranges. The first major critical variable was the concentration of the loading drug in the first water phase, which was largely limited by the inherent solubility at room temperature. In the case of aspirin, without solvents added, the maximum concentration was 3 mg/mL directly at the solubility limit. Runs at the 5–10 mg/mL range resulted in aggregation and lower quality. In the case of prednisolone, it was anticipated that on a per gram basis at least 1 mg/mL would be required to achieve high quality in addition to a realistic dosing paradigm for a rodent heart with target of 1 gram mass. This was achieved suitably with 10% Ethanol, however concentrations greater than 25% in an attempt to load more drug distorted the process (data not shown). The PLGA and PVA stabilizer system as presented here is therefore open to excipient manipulation provided that solubility and other attributes of the selected drug are addressed such to prevent deviations in overall quality which may or may not be desired depending on the application. We anticipate this platform would be open for further experimentation by professional formulation scientists tailored to each specific PLGA/drug selection for the intended direct injection application.

### In-vitro myocyte transfection

**Figure 5:**
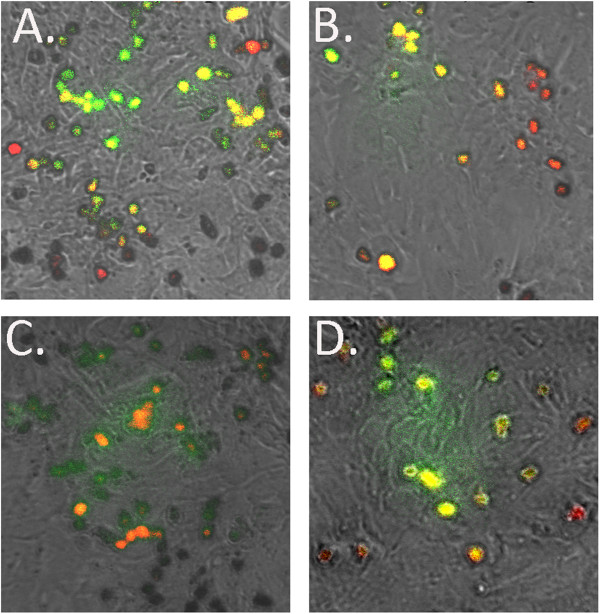
**In Vitro Fluorescent Imaging at 48 hours post transfection.** All 4 particle systems exhibited safe and robust uptake in myocytes while not interfering with plasmid uptake and subsequent GFP expression. Yellow signal indicates co-existence of GFP and nanoparticle in: **A**. PLGA65:35 Aspirin **B**. PLGA50:50 Aspirin **C**. PLGA50:50 Prednisolone **D**. PLGA65:35 Prednisolone.

All wells were checked for viability and it was determined that none indicated any major media discoloration or visual evidence of contamination. The following 5 groups all had positive detection of GFP (green) in at least 2/3 replicate wells at both 24 and 48 hours, with a greater degree of cells positive as expected at 48 hours. Figure 5 depicts independent uptake of both GFP plasmid and nanoparticle co-signal. The absorption clusters were confirmed in the center of myocytes. The multiple DNA and nanoparticle infection groups yielded nanoparticle presence (red) or both (yellow) at the 24 and 48 post transfection: I. Control GFP DNA only (data not shown) II. GFP and PLGA65:35 Aspirin (Figure 5A) III. GFP and PLGA50:50 Aspirin (Figure 5B) IV. GFP and PLGA50:50 Prednisolone (Figure 5C) V. GFP and PLGA65:35 Prednisolone (Figure 5D).

## Discussion

This study presents two key findings that have broad implications for the advancement of cardiac gene therapies. First a reproducible, simple to use lab scale process was developed to generate anti-inflammatory nanoparticles of very high quality for co-administration with gene products in a - regulatory friendly - PLGA platform. Although only two anti-inflammatory drugs were utilized in this feasibility assessment, it is anticipated that any other drug indicated for injection into muscle could be introduced by modification of the first drug water phase. Also the process offers an easy means to adjust the polymer content in the oil phase for the desired degradation/release profile, along with increasing the amount of stabilizer. Therefore this system can provide a platform to guide future pre-clinical studies to investigate reliable clinical interventions to address the role of inflammation on the relative performance of gene products in muscle tissue. The potential role of inflammation should not be overlooked, particularly in myocardial tissue where the most common delivery scenario is in ischemic regions, which are characterized by a high degree of inflammation and fibrosis. The second key finding in the final test was that PLGA uptake and release of anti-inflammatory agents in myocytes does not interfere with the absorption and trafficking of the GFP plasmid. Muscle tissue has a high risk of developing an adaptive immune response to gene products. Wilson et al. described in detail the host response after AAV delivery by route of administration and more specifically the role of inflammation [27]. A key finding with AAV mediated gene transfer was that the host either induces tolerance or an adaptive immune reaction through a series of complex interactions [28-30]. A prime risk factor in these interactions that was found to trigger adaptive immune responses were inflammatory cytokines and signals either already present in tissue or induced at the time of delivery [31]. It has been postulated that with attenuation of innate inflammatory response signals, the immune system has a much lower risk for mounting maladaptive T cell responses. Using the example of AAV, once vector capsid antigens are cleared from the system, typically 12–16 weeks after delivery, there is a good chance for therapeutic tolerance. The risk is that an adaptive immune response will destroy those cells expressing the transgene of interest well before these antigens are cleared. Use of anti-inflammatory agents to mitigate the innate response to injury is likely to result in enhanced long term gene expression. Intravenous delivery approaches are associated with a lower level of induced inflammation but are also very inefficient. In contrast, the IM route in the heart remains attractive because greater cardiac specificity can be achieved, especially for angiogenesis or regenerative therapies that require a more local delivery profile. Yet IM delivery is associated with a more robust innate immune response due to associated tissue injury.

Direct injection into healthy or ischemic myocardial regions introduces the gene product into a highly inflammatory region, which likely explains the poor cardiac gene therapy results with IM interventions. Early studies by Snyder et al. [32] reported that very little successful transfer occurs in damaged muscle in the inflammatory environment. Numerous examples have validated these observations in gene therapy trials. In hemophilia trials for example it was found that IM injection into skeletal muscle resulted in transient therapeutic gene expression and an adaptive CD4+ immune response [33,34]. However, delivery of the same product infused into the liver has resulted in better outcomes and limited reactions. Muscular dystrophy trials have encountered similar difficulties and have attempted to utilize immunosuppressant drugs and other agents to limit responses after multiple IM injections compromising patient safety [35].

## Conclusions

In this proof of concept study, GFP plasmid was utilized to simulate a therapeutic construct understanding that naked DNA is likely to be at the lower end in terms of transduction efficiency. For more practical gene therapy applications, it is anticipated that viral vectors encoding the gene of interest could readily be combined with particles containing potent anti-inflammatory drugs. The hypothesis offered is that with the right formulation, the anti-inflammatory agent would be released at the sufficient level over the critical post-delivery inflammatory period to provide an optimal viral vector trafficking microenvironment. There would be a predicted increase in transduction efficiency, minimize the innate and adaptive immune response to the vector and/or transgene and promote long term gene expression in the target tissues. This strategy of course would not be without its own limitations and would require much more experimentation to determine the best matched drug and release profile for co-administration into the heart. More complex approaches in managing the host response following therapy have been applied, however it may turn out that simply addressing the innate immune response at the time of delivery may be a meritorious approach to advance successful clinical translation.

## Competing interests

The authors declare that they have no competing interests.

## Authors’ contributions

ASF: Process development, particle evaluation, executed in vitro testing, wrote manuscript. AM: Performed myocyte harvest and quality control; study design input. MGK: Study design input, manuscript drafting and revisions. RDW: Quantitative sizing analysis, figure generation, manuscript editing. KBM: Study design input, myocyte evaluation, manuscript editing. DBW: GPF plasmid design and evaluation of results, manuscript editing. SY: Nanotechnology evaluations, experimental design, process development techniques, manuscript editing. CRB: Study design and manuscript editing. All authors read and approved the final manuscript.
